# Neonatal mortality and coverage of essential newborn interventions 2010 - 2013: a prospective, population-based study from low-middle income countries

**DOI:** 10.1186/1742-4755-12-S2-S6

**Published:** 2015-06-08

**Authors:** Sangappa M Dhaded, Manjunath S Somannavar, Sunil S Vernekar, Shivaprasad S Goudar, Musaku Mwenche, Richard Derman, Janet L Moore, Archana Patel, Omrana Pasha, Fabian Esamai, Ana Garces, Fernando Althabe, Elwyn Chomba, Edward A Liechty, K Michael Hambidge, Nancy F Krebs, Mabel Berrueta, Alvaro Ciganda, Patricia L Hibberd, Robert L Goldenberg, Elizabeth M McClure, Marion Koso-Thomas, Albert Manasyan, Waldemar A Carlo

**Affiliations:** 1 Women's and Children's Health Research Unit, KLE University's Jawaharlal Nehru Medical College, Belgaum, Karnataka, India; 2 University Teaching Hospital, University of Zambia, Lusaka, Zambia; 3 Christiana Care Health Services, Newark, DE, USA; 4 RTI International, Durham, NC, USA; 5 Indira Gandhi Government Medical College and Lata Medical Research Foundation, Nagpur, Maharashtra, India; 6 Department of Community Health Sciences, Aga Khan University, Karachi, Pakistan; 7 Moi University School of Medicine, Eldoret, Kenya; 8 Fundación para la Alimentación y Nutrición de Centro América y Panamá (FANCAP), Guatemala City, Guatemala; 9 Institute for Clinical Effectiveness and Health Policy, Buenos Aires, Argentina; 10 Indiana University School of Medicine, Indianapolis, IN, USA; 11 University of Colorado School of Medicine, Denver, CO, USA; 12 Massachusetts General Hospital for Children, Boston, MA, USA; 13 Department of Obstetrics/Gynecology, Columbia University, New York, NY, USA; 14 Eunice Kennedy Shriver National Institute of Child Health and Human Development, Bethesda, MD, USA; 15 University of Alabama at Birmingham, Birmingham, AL, USA

**Keywords:** neonatal mortality, newborn care, risk factors

## Abstract

**Background:**

Approximately 3 million neonatal deaths occur each year worldwide. Simple interventions have been tested and found to be effective in reducing the neonatal mortality. In order to effectively implement public health interventions, it is important to know the rates of neonatal mortality and understand the contributing risk factors. Hence, this prospective, population-based, observational study was carried out to inform these needs.

**Methods:**

The Global Network’s Maternal Newborn Health Registry was initiated in the seven sites in 2008. Registry administrators (RAs) attempt to identify and enroll all eligible women by 20 weeks gestation and collect basic health data, and outcomes after delivery and at 6 weeks post-partum. All study data were collected, reviewed, and edited by staff at each study site. The study was reviewed and approved by each sites’ ethics review committee.

**Results:**

Overall, the 7-day neonatal mortality rate (NMR) was 20.6 per 1000 live births and the 28-day NMR was 25.7 per 1000 live births. Higher neonatal mortality was associated with maternal age > 35 and <20 years relative to women 20-35 years of age. Preterm births were at increased risk of both early and 28-day neonatal mortality (RR 8.1, 95% CI 7.5-8.8 and 7.5, 95% CI 6.9-8.1) compared to term as were those with low birth weight (<2500g). Neonatal resuscitation rates were 4.8% for hospital deliveries compared to 0.9% for home births. In the hospital, 26.5% of deliveries were by cesarean section with an overall cesarean section rate of 12.5%. Neonatal mortality rates were highest in the Pakistan site and lowest in Argentina.

**Conclusions:**

Using prospectively collected data with high follow up rates (99%), we documented characteristics associated with neonatal mortality. Low birth weight and prematurity are among the strongest predictors of neonatal mortality. Other risk factors for neonatal deaths included male gender, multiple gestation and major congenital anomalies. Breech presentation/transverse lie, and no antenatal care were also significant risk factors for neonatal death. Coverage of interventions varied by setting of delivery, with the overall population rate of most evidence-based interventions low. This study informs about risk factors for neonatal mortality which can serve to design strategies/interventions to reduce risk of neonatal mortality.

**Trial registration:**

The trial is registered at clinicaltrials.gov. ClinicalTrial.gov Trial Registration: NCT01073475

## Background

Approximately 3 million neonatal deaths occur each year worldwide accounting for 40% of the under 5 mortality [1]. Three-quarters of neonatal deaths occur during the first week after birth, and of these nearly 75% occur in the first 24 hours. Worldwide, neonatal mortality has been reported to be caused by infection (36%), preterm birth (28%) and birth asphyxia (23%) [2-4]. Simple interventions aimed at these main causes have been tested and found to be effective in reducing the neonatal mortality [5,6].

First, to improve newborn care, emphasis has been placed on the delivery of all women within a health facility with capabilities to perform the essential obstetric and newborn care. Specifically, to reduce newborn mortality associated with birth asphyxia, access to high quality perinatal care, including cesarean section and newborn resuscitation, is needed [6-9]. To reduce mortality associated with preterm birth, evidence-based interventions including resuscitation care, skin-to-skin care, and exclusive breast feeding and support may be most effective. To reduce infection-related mortality, clean delivery practices, cord care, and treatment of possible infections are important. Estimates have suggested that more than 70% of newborn mortality is preventable with these existing evidence-based practices, but coverage of these interventions is low and uneven in geographic areas with highest burden of mortality [7]. Nearly half of women in low-resource areas still deliver outside health facilities, and many facilities are under-staffed or lack basic essential care [10,11].

Because the majority of deliveries occur in settings with poor health care systems, population-based rates of the coverage of these essential interventions have been lacking. Additionally, many reports on the trends of neonatal mortality use estimates or modeling and often do not provide precise estimates of the mortality rates [1,4]. Moreover, under-reporting of these vital events is more common in such settings, and estimates of mortality and availability of care are mainly obtained from hospital records and surveys [11,12].

In order to effectively implement public health interventions, the rates of neonatal mortality, the contributing factors, and the availability of the essential interventions across settings where women delivery are needed. Prospective studies may provide more specific data to inform these needs. However, these studies have been frequently small or restricted to hospital settings which limit their generalizability and ability to fully describe the trends [4].

Hence, there is a need for population-based, prospectively collected data on neonatal mortality, especially from low resource settings where most of the mortality occurs. This is essential for planning the interventions and prioritizing the health care delivery to improve newborn survival. To address this gap in knowledge, we undertook a prospective, population-based observational study of newborn outcomes in low resource settings of the Global Network for Women’s and Children’s Health Research (Global Network) using the Maternal Newborn Health Registry (MNHR) [13-15].

## Methods

The Global Network’s MNHR, a prospective, population-based, observational study, was initiated in 2008 and is an ongoing study being conducted at study sites in western Kenya, Zambia (Chongwe and Kafue), Pakistan (Thatta), India (Belgaum and Nagpur), Guatemala (Chimaltenango), and Argentina (Corrientes). Within each site, between 6 and 24 study clusters, geographic regions which were initiated with approximately 300 to 500 annual births were defined prospectively between 2008 and 2009. The objective of the MNHR is to include all pregnancies in these regions, regardless of delivery location [14].

Registry administrators (RAs) who are full time MNHR study staff aim to identify and enroll all eligible, pregnant women residing within the defined clusters by 20 weeks gestation, regardless of contact with the health care system or planned delivery location. After obtaining the woman’s consent, the RAs collected basic health data and conduct follow-up visits at two points: after delivery to collect outcomes at delivery and at 6 weeks post-partum. At the visit following each delivery, information on resuscitation with bag and mask, skin to skin contact after delivery, initiation of breastfeeding within one hour of birth, and bathing within six hours of birth was collected by interviewing the birth attendant and the mother/family members. In addition, basic obstetric practice including birth attendant type, and practices including administration of uterotonic agents during or immediately following delivery, administration of maternal antibiotics, and setting of delivery (hospital, clinic or health center or home, which included birth attendant’s home) were documented. Regardless of delivery setting, the RAs obtained the birth outcome and sought to interview the birth attendant (whether traditional or skilled) who assisted in the delivery. In case of neonatal death, RAs conducted a basic death audit by interviewing birth attendant and/or family members, including the mother whenever possible.

Neonatal mortality was evaluated both following delivery and at the 6-week follow-up visit. The neonatal mortality rate was calculated both as the early neonatal mortality rate (early NMR) defined as the number of early (0-6 days) neonatal deaths per 1,000 live births, and as the 28-day neonatal mortality rate (NMR), defined as the number of neonatal deaths (defined as deaths through 28 days of life) per 1000 live births.

## Data analyses

All study data were collected, reviewed, and edited by staff at each study site. Data were then transmitted to a central data coordinating center (RTI International, Durham, NC) using a secure process, with additional edits performed centrally and addressed at each site. Descriptive analyses included calculating the frequency and distribution of values. Generalized linear models were used to evaluate the relationship of potential risk factors [9-11] and early and 28-day neonatal mortality and to develop point and interval estimates of the relative risk associated with these risk factors. Finally, generalized estimating equations were used to account for the correlation of outcomes within cluster in developing appropriate p-values and confidence intervals. Data were analyzed using SAS v.9.3 (Cary, NC).

## Ethics approval

The MNHR study was reviewed and approved by each sites’ ethics review committee (Institute for Clinical Effectiveness and Health Policy, Argentina; San Carlos University, Guatemala; University of Zambia, Zambia; Moi University, Kenya; Aga Khan University, Pakistan; KLE University’s Jawaharlal Nehru Medical College; and Indira Gandhi Medical College, India), the U.S. partner’s institutional review boards, and the data coordinating center (RTI International). All women provided informed consent for data collection.

## Results

**Figure 1:**
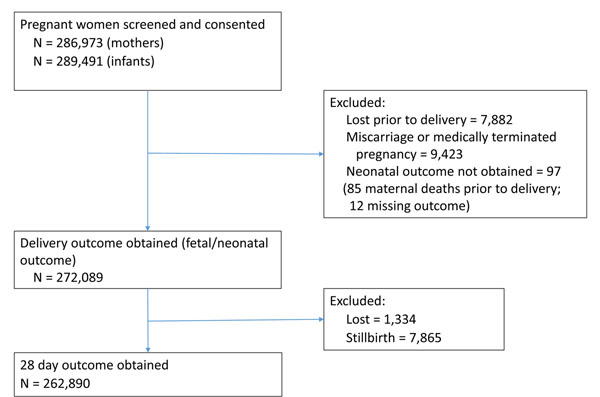
CONSORT Diagram for neonatal study, 2010 - 2013

During the study period from January 2010 through December 2013, a total of 269,614 women were enrolled, who had 262,890 live births (a total of 7,624 stillbirths were also reported which were excluded from subsequent analyses for this study) (Figure 1). Among live births, the follow-up rate at 6 weeks postpartum was 98.9%. For the entire study period, across all sites, the overall early NMR was 20.6 per 1000 live births and the 28-day NMR was 25.7 per 1000 live births.

Table 1 describes maternal characteristics and risks for early and 28-day neonatal mortality. Higher neonatal mortality was associated with maternal age > 35 and <20 years relative to women 20-35 years of age. Among women with no formal education, the risk of early NMR was also significantly higher (RR 1.6, CI 1.4- 1.9), relative to those with primary school or higher education. Additionally, no antenatal care (ANC) was associated with higher risk of early neonatal mortality, and among women receiving ANC, having fewer visits was associated with increasing risk of both early and 28-day neonatal mortality.

**Table 1:** Early and 28-day neonatal mortality and maternal characteristics for registry, 2010-2013

	Alive at Day 28 N (%)*	Early Neonatal Death N (%)*	28-day Neonatal Death N (%)*	RR (95% CI)** for Early NMR	RR (95% CI)** for 28-Day NMR
Maternal age					

< 20	30,953 (12.1)	615 (11.4)	758 (11.2)	1.2 (1.1, 1.3)	1.2 (1.1, 1.3)

20-35	215,011 (84.1)	4,527 (83.8)	5,657 (83.8)	1.0	1.0

> 35	9,754 (3.8)	259 (4.8)	338 (5.0)	1.3 (1.1, 1.5)	1.3 (1.2, 1.5)

Maternal education					

No formal education	61,649 (24.2)	2,073 (38.5)	2,628 (39.0)	1.6 (1.4, 1.9)	1.7 (1.5, 2.0)

Primary	97,912 (38.4)	1,717 (31.9)	2,146 (31.8)	1.4 (1.2, 1.6)	1.5 (1.3, 1.6)

Secondary	77,011 (30.2)	1,310 (24.3)	1,632 (24.2)	1.2 (1.0, 1.4)	1.3 (1.1, 1.4)

University+	18,381 (7.2)	287 (5.3)	332 (4.9)	1.0	1.0

Parity	255,381	5,387	6,736		

0	85,581 (33.5)	2,067 (38.4)	2,527 (37.5)	1.4 (1.3, 1.5)	1.4 (1.3, 1.5)

1-2	108,556 (42.5)	1,852 (34.4)	2,317 (34.4)	1.0	1.0

> 2	61,244 (24.0)	1,468 (27.3)	1,892 (28.1)	1.2 (1.1, 1.3)	1.2 (1.1, 1.3)

At least one ANC visit					

Yes	247,027 (96.7)	5,044 (93.6)	6,274 (93.1)	1.0	1.0

No	8,544 (3.3)	345 (6.4)	462 (6.9)	1.3 (1.0, 1.7)	1.4 (1.1, 1.8)

ANC visits					

0	3,455 (2.5)	139 (4.8)	194 (5.4)	1.4 (1.0, 1.8)	1.6 (1.3, 1.9)

1-2	24,992 (18.1)	754 (26.2)	937 (25.9)	1.3 (1.1, 1.4)	1.3 (1.1, 1.4)

≥ 3	109,660 (79.4)	1,988 (69.0)	2,493 (68.8)	1.0	1.0

*N’s less than the total represent missing responses

**Relative risk (RR) and 95% CI use GEE to account for study cluster

We also assessed the newborn characteristics associated with early and 28-day neonatal mortality. The preterm births accounted for 44.6% (n = 2,172) of the early neonatal deaths and 42.8% (n=2,615) of the 28-day deaths (Table 2). The NMR was 114.7 per 1000 live births for preterm births compared to 15.0 per 1000 for term births and similarly was 121.7 per 1000 for those <2500 g vs. 13.6 per 1000 for ≥2500 g births. Among those born preterm, risk of both early and 28-day neonatal deaths (RR 8.1, 95% CI 7.5, 8.8 and RR 7.5, 95% CI 6.9, 8.1) was higher relative to term births. Those born with low birth weight (<2500g) were also at increased risk of neonatal mortality relative to those born with birth weight ≥2500 g, RR 5.7 (95% CI 5.1, 6.3 of neonatal mortality for those 1500-2499 vs. ≥2500 g). Male infants were at increased risk (RR 1.2, 95% CI 1.1, 1.2 vs. female) and as were those from multiple gestations (RR 6.6, 95% CI 6.0, 7.3 vs. singleton). Those neonates with major congenital anomalies had higher risk of 28-day neonatal death (RR 14.6, 95% CI 12.4, 17.3). Finally, women with a breech presentation also had higher risk of neonatal death (RR 2.8, CI 2.4, 3.2) compared to women with a non-breech presentation.

**Table 2:** Early and 28-day neonatal mortality and newborn characteristics for registry, 2010-2013*

	Alive at Day 28 N (%)*	Early Neonatal Death N (%)*	28-Day Death N (%)*	RR (95% CI)** for Early NMR	RR (95% CI)** for 28-Day NMR
Births, N	256,126	5,412	6,764	--	--

Estimated gestational age					

Preterm	20,174 (8.1)	2,172 (44.6)	2,615 (42.8)	8.1 (7.5, 8.8)	7.5 (6.9, 8.1)

Term	228,935 (91.9)	2,703 (55.4)	3,492 (57.2)	1.0	1.0

Birth weight category					

< 1000g	52 (0.0)	286 (5.3)	315 (4.7)	71.8 (63.2, 81.5)	61.4 (54.8, 68.9)

1000-1499g	639 (0.2)	812 (15.1)	970 (14.4)	46.0 (41.0, 51.6)	42.7 (38.2, 47.8)

1500-2499g	25,581 (10.0)	1,784 (33.2)	2,259 (33.6)	5.7 (5.1, 6.4)	5.7 (5.1, 6.3)

≥ 2500g	229,732 (89.7)	2,485 (46.3)	3,174 (47.2)	1.0	1.0

Infant gender					

Male	132,424 (51.7)	3,099 (57.4)	3,780 (56.0)	1.2 (1.2, 1.3)	1.2 (1.1, 1.2)

Female	123,653 (48.3)	2,303 (42.6)	2,973 (44.0)	1.0	1.0

Multiple birth					

Yes	3,693 (1.4)	567 (10.5)	704 (10.4)	6.7 (6.0, 7.6)	6.6 (6.0, 7.3)

No	252,352 (98.6)	4,838 (89.5)	6,052 (89.6)	1.0	1.0

Congenital anomaly					

Present	695 (0.3)	318 (6.0)	392 (5.9)	15.0 (12.5, 17.9)	14.6 (12.4, 17.3)

Absent	251,599 (99.7)	4,952 (94.0)	6,207 (94.1)	1.0	1.0

Breech presentation					

Yes	5,285 (2.1)	353 (6.5)	423 (6.3)	3.0 (2.5, 3.4)	2.8 (2.4, 3.2)

No				1.0	1.0

*N’s less than the total represent missing responses

**Relative risk (RR) and 95% CI use GEE to account for study cluster

Next, we assessed the rates of essential care practices by location of delivery. Altogether, 46.2% of the deliveries occurred at a hospital, 25.5% at a clinic and 28.4% at home or birth attendant’s home (data not shown). Among deliveries occurring at hospitals, 59.3% of women received uterotonic agents during or following delivery, compared to 47% of those delivering at a clinic and 4.0% of those delivering in a home setting (Table 3). Similarly, 60.7% of those women delivering in a hospital received antibiotics, compared to 37.1% at a clinic and 2.2% at home. Regarding essential newborn practices, 4.8% of those delivered at hospitals were resuscitated, compared to 3.8% of those in clinic and 0.9% delivered in a home setting. Breastfeeding rates were similar between the settings, with 72.5% of those in hospitals, 81.7% of those in clinics and 69.0% of those in home settings reporting breastfeeding within one hour of delivery. Early skin-to-skin contact rates were higher at the hospital (32.5%) and health center (25.0%) compared to home (12.1%). Early bathing, a practice not recommended, was reported at lower rates at the hospital (7.9%) compared to clinics (13.3%) and home (40.0%). Finally, use of cord care was higher at hospitals (44.8%) compared to clinics (30.2%) and home (23.8%). Among hospital births, 26.5% of the deliveries were performed by cesarean section for an overall rate of 12.5% in the population (data not shown).

**Table 3:** Rates of interventions by delivery location for registry deliveries, 2010- 2013

	Delivery Location		
	Hospital	Clinic	Home/Other

**Deliveries, N**	119,994	66,554	74,091

Uterotonics, N (%)	70,144 (59.3)	31,137 (47.0)	2,956 (4.0)

Maternal antibiotics, N (%)	72,026 (60.7)	24,596 (37.1)	1,591 (2.2)

			

**Live births, N**	121,291	66,952	74,573

Bag and mask resuscitation, N (%)	5,696 (4.8)	2,511 (3.8)	702 (0.9)

Breastfeed within 1 hour, N (%)	86,098 (72.5)	54,163 (81.7)	51,040 (69.0)

Skin-to-skin contact, N (%)	39,030 (32.5)	16,680 (25.0)	8,877 (12.1)

Bathed within 6 hours, N (%)	9,287 (7.9)	8,811 (13.3)	29,637 (40.0)

Cord care, N (%)	54,007 (44.8)	20,165 (30.2)	17,432 (23.8)

**Figure 2:**
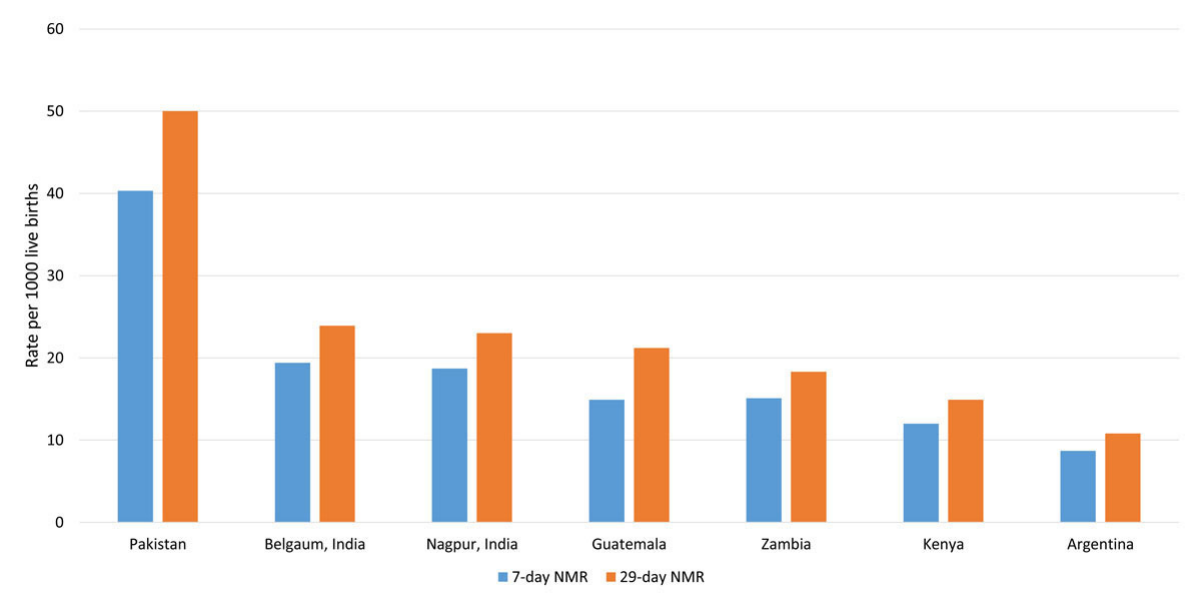
Early and 28-day neonatal mortality by study site, 2010-2013

Finally, we evaluated the characteristics by site. Figure 2 summarizes the early and 28-day mortality by site. Rates were highest in the Pakistan site (40.3/1000 and 50.0/1000 for early and 28-day neonatal mortality, respectively) and lowest in Argentina. When we evaluated the obstetric and essential newborn care practices by site (Table 4), starting of breastfeeding within one hour of delivery ranged from 92% in Zambia to 23% in Pakistan. Initiation of skin-to-skin contact was 83% in Argentina and in rest of the sites, ranged from 31% in Nagpur to 2% in Pakistan. 99% of the babies did not receive a bath in the first six hours of birth in Nagpur and Argentina and 48% of them did not receive a bath in Guatemala. Regarding obstetric care, cesarean section rates ranged from 1.1% in Zambia to 34.9% in Argentina and the use of oxytocics ranged from 17.5% in Zambia to 96.1% in Argentina. Finally, maternal antibiotic use ranged from 1.2% in Zambia to 86.4% in Nagpur, India.

**Table 4:** Obstetric and Essential Care Practices by Global Network Site, 2010-2013

	Africa		Asia			Latin America	
	
	Kenya, N (%)	Zambia, N (%)	Belgaum, N (%)	Nagpur, N (%)	Pakistan, N (%)	Argentina, N (%)	Guatemala, N (%)
**Deliveries, N**	34,878	26,791	77,649	38,070	44,933	9,716	28,675

Cesarean section	493 (1.4)	286 (1.1)	11,097 (14.3)	7,601 (20.0)	4,293 (9.6)	3,393 (34.9)	5,348 (18.7)

Uterotonics	9,548 (27.5)	4,667 (17.5)	29,239 (38.1)	27,871 (73.3)	15,595 (34.8)	9,314 (96.1)	7,987 (28.6)

Maternal antibiotics	2,666 (7.7)	330 (1.2)	37,368 (48.5)	32,792 (86.4)	13,509 (30.1)	5,591 (57.7)	5,975 (21.3)

							

**Live births, N**	35,303	27,010	78,190	38,327	45,427	9,774	28,859

Bag and mask resuscitation	595 (1.7)	531 (2.0)	3,344 (4.3)	1,312 (3.5)	2,410 (5.3)	406 (4.2)	319 (1.1)

Breastfeed within 1 hour	28,937 (82.1)	24,623 (91.6)	63,927 (85.2)	32,797 (86.0)	10,597 (23.4)	8,633 (89.0)	21,823 (75.9)

Skin-to-skin contact	8,787 (25.1)	6,235 (23.4)	22,424 (29.0)	11,847 (31.1)	955 (2.1)	7,911 (82.6)	6,432 (22.6)

Bathed within 6 hours	15,515 (44.0)	5,441 (20.2)	3,681 (4.8)	340 (0.9)	8,944 (19.7)	87 (0.9)	13,739 (52.2)

## Discussion

The Global Network’s MNHR, a prospective, observational study, is being conducted in seven sites with the aim of providing reliable, community-based data on rates of maternal, fetal and neonatal mortality as well as factors that may be related to these outcomes. Designated field staff obtain health outcomes with their work monitored by central study staff. One of the strengths of the study was that nearly all women residing within the cluster for delivery were enrolled and very few of the women or infants were lost to follow-up [16]. Thus, the MNHR documents both mortality rates as well as coverage of interventions across all delivery locations for the study regions.

Both the early and 28-day neonatal mortality rates varied profoundly between the study sites, with the highest 28-day rates of 50 per 1000 live births reported in Pakistan and the lowest rates observed in the Argentina clusters. These rates were similar to the estimates from the Global Burden of Disease study (2013) [17]. We also observed differences in indicators of both obstetric and newborn care across sites.

As expected, neonatal deaths were more common in preterm and in low birth weight babies, with more than 40% of the early and 28-day neonatal deaths accounted for by those born preterm or low birth weight [18-20]. Other risk factors for neonatal deaths included male gender and those from a multiple gestation. Major congenital anomalies and breech presentation or transverse lie were also significant risk factors for neonatal death. Similar observations were noted in the *Lancet* Neonatal Survival Series 1 (2005) [5]. Women who had no antenatal care were at increased risk of experiencing a neonatal death as were women who were without any formal care at the time of delivery.

The coverage of evidence-based interventions varied widely by setting of delivery, as well as by study site. Overall, with the exception of breastfeeding rates which were similar across settings, hospital deliveries had improved coverage of the essential newborn care as well as obstetric interventions, compared to clinic or home settings. For example, it is estimated that 5% of all deliveries require basic resuscitation, approximately the rate observed in hospitals [21,22], but the population-rate was much lower. Overall there was uneven coverage of these evidence-based interventions. For example, while there was a cesarean section rate of over 25% among facility-deliveries, this translated to 12.5% among the entire population. When we examined coverage by site, the disparity was clear, with only 1% cesarean section rates at the African sites.

A prior study to evaluate availability of essential interventions in the health facilities serving the MNHR clusters found low coverage of basic equipment and medicines with few facilities having physicians available 24 hours/day, seven days a week [10]. As international organizations have placed an emphasis on increasing facility-based deliveries in order to improve birth outcomes [8], access to antenatal care including birth preparedness with timely transport, as well quality of obstetric and neonatal care in these settings will be needed to help reduce neonatal mortality [7,8,23].

Further research is needed to define which interventions will result in substantial reductions in neonatal mortality and in particular, those interventions that may be effective in reducing mortality associated with preterm birth. Additionally, the disparity in coverage of interventions was clear. Women who did not deliver at a health facility had much lower access to essential care.

## Conclusions

Using prospectively collected data with high follow up rates (99%), we have documented characteristics that are associated with neonatal mortality. Low birth weight and prematurity are among the strongest predictors of neonatal mortality. Congenital anomaly is also highly associated. These data support the recent evidence that prematurity and low birth weight are now the most common causes of neonatal mortality worldwide [20]. Several maternal and delivery characteristics are also associated with high neonatal mortality risk. This study informs about risk factors for neonatal mortality which can serve to design strategies for decreasing neonatal mortality. Furthermore, it is clear that women who delivered outside a health facility were much less likely to receive essential obstetric and newborn care. Ensuring women have access to this basic care is an important step to reducing newborn mortality.

## List of abbreviations used

MNHR: maternal newborn health registry; NMR: neonatal mortality rate; RA: registry administrator; TBA: traditional birth attendant.

## Competing interests

The authors declare they have no competing interests.

Authors’ contributions: SMD conceived the idea for this study. SMD, MSS, WAC and EMM wrote the first draft. JLM and EMM performed data analyses. SMD, MSS, SSV, SSG, MW, RD, JLM, AP, OP, FE, AG, FA, EC, EAL, KMH, NFK, MB, AC, PLH, RLG, EMM, MKT, AM, WAC participated in study design study site oversight and data collection. All authors read and approved the final manuscript

## Peer review

Reviewer reports for this article can be found in Additional file 1.

## Supplementary Material

Additional file 1 Click here for file

## Declarations

This article has been published as part of *Reproductive Health* Volume 12 Supplement 2, 2015: Research reports from the NICHD Global Network for Women’s and Children’s Health Research Maternal and Newborn Health Registry. The full contents of the supplement are available online at http://www.reproductive-health-journal.com/supplements/12/S2. Publication of this supplement was supported by grants from the Eunice Kennedy Shriver National Institute of Child Health and Human Development to RTI International.
