# Supplementary material for: Neonatal mortality and coverage of essential newborn interventions 2010 - 2013: a prospective, population-based study from low-middle income countries
Source: Reprod Health. 2015 Jun 8;12(Suppl 2):S6. doi: 10.1186/1742-4755-12-S2-S6 (PMC4464215; doi:10.1186/1742-4755-12-S2-S6)
Supplement: Additional file 1 [file 1742-4755-12-S2-S6-S1.pdf]

Referee's comments to the authors– this sheet <u>WILL</u> be seen by the author(s) and published with the article

Title	Neonatal Mortality and Risk Factors 2010 - 2013: A prospective, population-based Global Network Study
Author(s)	Sangappa M Dhaded, Manjunath S Somannavar, Sunil S Vernekar, Shivaprasad S Goudar, Musaku Mwenche, Richard Derman, Janet L Moore, Archana Patel, Omrana Pasha, Fabian Esamai, Ana Garces, Fernando Althabe, Elwyn Chomba, Edward A Liechty, K Michael Hambidge, Nancy F Krebs, Mabel Berrueta, Alvaro Ciganda, Patricia L Hibberd, Robert L Goldenberg, Elizabeth M McClure, Marion Koso-Thomas, Albert Manasyan, and Waldemar A Carlo
Referee's name	Beena Kamath-Rayne

When assessing the work, please consider the following points, where applicable:

1. Is the question posed by the authors new and well defined?
2. Are the methods appropriate and well described, and are sufficient details provided to replicate the work?
3. Are the data sound and well controlled?
4. Does the manuscript adhere to the relevant standards for reporting and data deposition?
5. Are the discussion and conclusions well balanced and adequately supported by the data?
6. Do the title and abstract accurately convey what has been found?
7. Is the writing acceptable?

Please make your report as constructive and detailed as possible in your comments so that authors have the opportunity to overcome any serious deficiencies that you find and please also divide your comments into the following categories:

- Major Compulsory Revisions (which the author must respond to before a decision on publication can be reached)
- Minor Essential Revisions (such as missing labels on figures, or the wrong use of a term, which the author can be trusted to correct)
- Discretionary Revisions (which are recommendations for improvement but which the author can choose to ignore)

Where possible please supply references to substantiate your comments.

When referring to the manuscript please provide specific page and paragraph citations where appropriate.

General comments: Neonatal mortality is now an increasing proportion of overall child mortality, and studies are needed to understand the underlying causes of neonatal mortality, particularly in low and middle income countries, and particularly in rural areas where home births predominate. This manuscript describes an analysis of the Global Network for Women's and Children's Health Research using the Maternal Newborn Health Registry, spanning 2010 to 2013, that takes a prospective, population based approach towards describing risk factors and interventions that could impact neonatal mortality. Their data were collected from over 260,000 live births in clusters of 6 countries that had approximately 300-500 annual births per cluster. Eligible pregnant women were enrolled by 20 weeks gestation and followed until 6 weeks postpartum to collect data for the study. Neonatal mortality rate, including an early neonatal mortality rate, was calculated, and death audits were performed for all newborn deaths. The study is well written and makes an important contribution to our understanding of neonatal mortality in low- and middle-income countries where the majority of neonatal deaths are occurring.

Not surprisingly, the results of this study indicate that lack of antenatal care was associated with increased risk of early neonatal mortality, and the highest risk of mortality was associated with women who did not have a birth attendant at delivery. Prematurity, low-birth weight and congenital anomalies also increased the risk of both early and 28-day neonatal mortality. There were differences in neonatal mortality across sites as well as newborn and obstetric care practices.

Major compulsory revisions:

It is surprising, however, that decreased risk of early neonatal mortality was seen in deliveries occurring in a clinic or home-based setting, compared to a hospital based setting. This finding conflicts with recommendations for women to have facility-based births, with greater availability of emergency obstetric care and providers that can perform effective neonatal resuscitation. The authors comment in the Discussion that further investigation is needed in the quality of obstetric and neonatal care in facilities to reduce neonatal mortality. This is an important point that needs further emphasis, and also a mention in the abstract, so that the interpretation of the results is not that facility based births should not be encouraged. A helpful reference to support these assertions is Dickson, et al., and the Lancet Every Newborn Study Group, Lancet, 2014 that describes some of the health systems bottlenecks and strategies to improve quality universal maternal and neonatal coverage in low- and middle-income countries.

Another surprising finding is that babies who were bathed within 6 hours of age had a higher survival rate at Day 28 compared to those that were not; however, the mothers who practice skin to skin contact had babies that had higher rates of survival at Day 28 as well. In Essential Care for Every Baby, the teaching is that babies should not be bathed before 6 hours of age: being bathed before 6 hours is a risk factor for hypothermia. One weakness of the manuscript is that data are not presented about the temperatures of the babies who died, or whether temperatures were monitored regularly. This should be mentioned in the discussion, as well as bathing before 6 hours may be a proxy for birth attendance.

The differences in the performance of bag-mask ventilation are also worth further mention. Multiple studies have shown that with improvement in the initial steps of resuscitation, more babies will respond with spontaneous breathing and fewer will progress to needing bag-mask ventilation. The authors rightfully state that higher-risk deliveries are likely born in facility settings, and these may be at higher risk for needing resuscitation and overall mortality.

Discretionary revisions: In the Methods section, it would be helpful to describe the differences in how pregnant women were followed if they delivered at home versus in a facility-based setting, and make it clear that all women were able to be followed, no matter where they were delivered. If the women chose to deliver at home and accurate data were able to be collected, that is a strength of this study that should be emphasized.

Minor essential revisions:
I am unfamiliar with the abbreviation MTP in Figure 1.

Overall, I do think the authors have presented important information from a valuable research network that contributes to our understanding of neonatal mortality in low- and middle-income based settings. I think the authors could justify some of the findings based on my comments above so that readers of the manuscript can gain a better understanding of where some of the gaps in quality and health care coverage may play a role.

Referee's comments to the authors– this sheet WILL be seen by the author(s) and published with the article

Title	**Neonatal Mortality and Risk Factors 2010 - 2013: A prospective, population-based Global Network Study**
Author(s)	Sangappa M Dhaded[1],
Referee's name	Jose Belizan

When assessing the work, please consider the following points, where applicable:

1. Is the question posed by the authors new and well defined?
2. Are the methods appropriate and well described, and are sufficient details provided to replicate the work?
3. Are the data sound and well controlled?
4. Does the manuscript adhere to the relevant standards for reporting and data deposition?
5. Are the discussion and conclusions well balanced and adequately supported by the data?
6. Do the title and abstract accurately convey what has been found?
7. Is the writing acceptable?

Please make your report as constructive and detailed as possible in your comments so that authors have the opportunity to overcome any serious deficiencies that you find and please also divide your comments into the following categories:

- Major Compulsory Revisions (which the author must respond to before a decision on publication can be reached)
- Minor Essential Revisions (such as missing labels on figures, or the wrong use of a term, which the author can be trusted to correct)
- Discretionary Revisions (which are recommendations for improvement but which the author can choose to ignore)

Where possible please supply references to substantiate your comments.

When referring to the manuscript please provide specific page and paragraph citations where appropriate.

General comments:

Major compulsory revisions: XXX

Minor essential revisions:

Discretionary revisions:

(continue on the next sheet)

Continued:

Major concerns.
This paper in the current format is not giving major contribution to already published information. One relevant contribution is that it involves populations and communities but these characteristics are not clearly stated and reinforced in the text. The article should then reinforce these characteristics all over the text and in the title.

Another contribution of interest is the use of essential interventions by site and probably this subject could be further reinforced in the background, discussion and conclusion sections.

There is a mix of factors that could be risk factors for neonatal death with factors that can be a consequence of a death or linked to the poor health status previous to a death. Consequently factors like bag and mask resuscitation, breastfeeding within one hour, skin-to-skin, bathed within 6 hours and similar should be withdrawn from the analysis.

Also analysis of site of attention and similar should be withdrawn from the analysis since it is not a risk factor and the association could be derived from the referral of severe conditions.

In sum, the analysis of risk factors are of poor interest meanwhile the description of the use of essential interventions is of greater interest and the main focus of the paper on this topic will enhance the interest of the paper.

Minor concerns.

What means Oxytocics? Oxytocics during labour?, postpartum? Please describe.

Regarding figure 2, what about putting the countries in order of the mortality rate starting by the lowest one?

Supplement Editor comments:

We consider that the paper needs major revision before being able to be considered for publication.
